# Impact of early vs delayed initiation of dual antimicrobial salvage therapy on clinical outcomes in MRSA bacteremia

**DOI:** 10.1128/spectrum.00442-26

**Published:** 2026-06-30

**Authors:** Victoria Sanderford, Nikko Rowe A. Tabliago, Sarah Alnami, Susan Spencer, Rachel Colven, Gustavo Rey Alvira-Arill, Zachary Gruss, Laura Ehrenberger, Aaron Hamby, Abby Kosharek, Stephen Thacker, Krutika Mediwala Hornback, Taylor Morrisette, Richard Lueking

**Affiliations:** 1 Department of Medicine, Division of Internal Medicine, Medical University of South Carolina 2345, Charleston, South Carolina, USA; 2 Department of Medicine, Division of Infectious Diseases, Medical University of South Carolina Health 158156, Charleston, South Carolina, USA; 3 Department of Pharmacy Services, Medical University of South Carolina Health 158156, Charleston, South Carolina, USA; 4 Department of Clinical Pharmacy & Outcomes Sciences, Medical University of South Carolina College of Pharmacy 15472, Charleston, South Carolina, USA; 5 Department of Pediatrics, Division of Infectious Diseases, Medical University of South Carolina Health 158156, Charleston, South Carolina, USA; University of Calgary, Calgary, Alberta, Canada

**Keywords:** MRSA, bacteremia, salvage therapy, ceftaroline

## Abstract

Our retrospective observational cohort study investigated the effect of the timing of escalation to dual salvage therapy utilizing ceftaroline with vancomycin or daptomycin for the treatment of persistent methicillin-resistant *Staphylococcus aureus* bacteremia on patient outcomes. Eligibility criteria included age ≥18 years old, at least one positive blood culture for methicillin-resistant *Staphylococcus aureus*, and receipt of salvage therapy consisting of ceftaroline in combination with either vancomycin or daptomycin for any duration during their bloodstream infection within our health system over a 10-year period. This resulted in the inclusion of 189 adult patients. Patients were stratified by the timing of ceftaroline initiation: ≤5 days (early initiation) versus >5 days (late initiation) from index positive culture. The study populations were similar, although there was a higher prevalence of persons who inject drugs started on early salvage therapy (36.0% versus 18.0%, *P* = 0.005) and fewer patients with type II diabetes (25.8% versus 41.0%, *P* = 0.028). Analysis revealed no statistically significant differences between 90-day all-cause mortality or readmission rates in either group; however, there was less 90-day microbiologic recurrence in the early initiation group (3.4% versus 11.0%, *P* = 0.046). Patients started on early salvage therapy also had fewer total days of bacteremia (7.0 versus 10.5 days, *P* < 0.001), fewer metastatic foci of infection (61.8% versus 82.0%, *P* = 0.002), and a shorter duration of treatment (46.0 versus 50.0 days, *P* = 0.013).

**IMPORTANCE:**

Treatment of methicillin-resistant *Staphylococcus aureus* bacteremia remains a challenge due to its high morbidity and mortality. Ceftaroline has been studied for off-label utilization in dual salvage therapy with daptomycin or vancomycin in these patients, although current data regarding the optimal timing for its addition are limited. We sought to address this knowledge gap with our retrospective review of patient outcomes at our hospital system who received ceftaroline for this indication. Overall, earlier initiation of ceftaroline-based dual therapy was beneficial, as evidenced by reduced microbiologic recurrence, shorter duration of bacteremia, reduced metastatic disease, and subsequent shorter duration of therapy if patients were escalated to dual salvage therapy with ceftaroline within 5 days of the index culture.

## INTRODUCTION

Treatment of methicillin-resistant *Staphylococcus aureus* (MRSA) bacteremia remains a major clinical challenge because of its high rates of morbidity and mortality (1–3). Each additional day of persistent *Staphylococcus aureus* bloodstream infection (BSI) has been shown to increase mortality risk by 16–20%, and patients with MRSA BSI exhibit a threefold increased likelihood of prolonged infection compared to those with methicillin-susceptible isolates. Ninety-day mortality rates in MRSA BSI have been reported to be as high as 39% (1, 3–6). Furthermore, persistent BSI has been associated with increased risk of metastatic foci and worse overall clinical outcomes (3, 4, 7).

The Infectious Diseases Society of America (IDSA) describes persistent MRSA BSI as failure to achieve blood culture clearance within 7 days of index culture despite appropriate antimicrobial treatment. IDSA guidelines recommend consideration of an alternative regimen after 7 days of persistent bacteremia. One suggested regimen includes high-dose daptomycin (10 mg/kg/day, if susceptible) in combination with a second agent such as a β-lactam (8). Different from most other β-lactam agents, the fifth-generation cephalosporin ceftaroline has activity against MRSA through its affinity for the mecA-encoded penicillin-binding protein 2a and has demonstrated *in vitro* synergy when combined with vancomycin and daptomycin (9, 10). Currently, its use in MRSA bacteremia remains an off-label indication (11, 12).

Several previous studies have demonstrated the potential benefit of ceftaroline-based combination therapy in MRSA BSI, with lower mortality rates reported compared with monotherapy (13, 14). Geriak et al. reported a 0% versus 26% in-hospital mortality rate in the daptomycin-plus-ceftaroline combination therapy and monotherapy groups, respectively, when combination therapy was initiated within 72 h of MRSA bacteremia, prompting early termination of the study due to ethical concerns (13). The mortality benefit seen with receipt of dual therapy within 72 h of bacteremia onset suggests early transition to combination therapy may confer advantages beyond current guideline recommendations of escalating therapy at 7 days of persistent BSI (8, 13). This was further evidenced by separate research showing a correlation between time to transition from monotherapy to daptomycin-based dual therapy and the duration of bacteremia, although the average time of escalation to dual therapy was day 6 in this study (14). Although outcomes have been variable with ceftaroline-vancomycin combination therapy, one study reported a microbiologic cure rate of nearly 97%, suggesting that it is another reasonable salvage regimen (15–17).

Although previous work has examined the efficacy of ceftaroline-based combination therapy, the optimal timing of its initiation remains uncertain. The goal of our study was to directly compare an early intervention group to those who received ceftaroline-based dual therapy later in their bacteremia course, with the hypothesis that earlier initiation would result in improved clinical outcomes. Five days was chosen as a midpoint between existing data involving initiation of salvage therapy as early as day 3 of MRSA BSI and the current guideline-recommended timeline of 7 days (8, 13, 14). To address this knowledge gap, we evaluated the impact of early versus later initiation of ceftaroline combination therapy (≤5 days vs >5 days from index blood culture) on clinical outcomes, including 90-day mortality, bacteremia recurrence, and hospital readmission in patients with MRSA bacteremia.

## MATERIALS AND METHODS

### Study design and setting

We conducted a retrospective observational cohort study of adult patients with MRSA bacteremia treated across the Medical University of South Carolina (MUSC) Health system between January 1, 2014, and June 30, 2024. MUSC Health is a large academic health system with the flagship hospital located in Charleston, South Carolina, and nine satellite facilities. The primary objective was to compare clinical outcomes associated with ceftaroline-based combination salvage therapy with vancomycin or daptomycin when ceftaroline was added within ≤5 days (early initiation) versus >5 days (late initiation) of bacteremia onset. An additional exploratory analysis using ≤3 days versus >3 days as a threshold for early initiation was conducted to generate hypotheses for future investigation.

### Study population

Eligible patients were ≥18 years of age, had at least one positive blood culture for MRSA, and received salvage therapy consisting of ceftaroline in combination with either vancomycin or daptomycin for any duration during their MRSA BSI. Patients were excluded if salvage therapy was administered for indications other than MRSA BSI, if combination therapy was initiated after documented clearance of blood cultures, or if clinical data necessary for evaluating primary outcomes were missing or incomplete.

### Data collection

Included patients were extracted from electronic health records (EHR) using SlicerDicer within the Epic EHR system based on ceftaroline orders placed during the study period. Variables collected via chart review included patient demographics, common comorbidities, renal function, bacteremia and microbiologic characteristics, treatment characteristics including initial ceftaroline-based dual therapy regimen selected and whether they later required transition to an alternative salvage therapy, the presence of any adverse drug reactions, patient acuity as represented by the need for ICU-level care and a Pitt bacteremia score, source control interventions, and infectious diseases consult service involvement in their care. Study data were collected and managed using REDCap electronic data capture tools at MUSC (18, 19).

### Study definitions

The Pitt bacteremia severity score is a validated tool used to assess illness severity and predict mortality risk in patients with bloodstream infections. It accounts for patient temperature, mechanical ventilation requirements, cardiac arrest, mental status, and the presence of hypotension and is calculated within 48 h of the index positive blood culture. A score of ≥4 indicates a higher risk of mortality (20–22). The time to culture clearance was calculated from the index blood culture date to the date of the first negative blood culture. Source control refers to any intervention undertaken to eliminate an ongoing infection, such as removal of an infected intravenous line, surgical drainage, or device removal.

### Study endpoints

The primary endpoints included 90-day all-cause mortality, microbiologic recurrence of MRSA bacteremia, and all-cause hospital readmission. Secondary outcomes included time from initiation of ceftaroline-based combination therapy to blood culture clearance, total duration of bacteremia from index culture to clearance, total duration of MRSA-directed therapy, and metastatic complications (i.e., vertebral osteomyelitis, discitis, non-vertebral osteomyelitis, epidural abscess, septic arthritis, prosthetic joint infection, endophthalmitis, infective endocarditis/cardiac abscess, brain abscess, meningitis, cerebral septic emboli, pulmonary abscess, empyema, parapneumonic effusion, pulmonary septic emboli, splenic abscess, and renal abscess). Exploratory analyses were performed to evaluate the association between the timing of salvage therapy initiation and clinical outcomes. Specifically, the primary endpoint was compared between patients who received ceftaroline-based combination salvage therapy within ≤5 days and >5 days from the index blood culture. Additional analyses were performed to compare the primary outcomes between patients receiving combination therapy within ≤3 days and >3 days of bacteremia onset.

### Statistical analysis

Descriptive statistics were reported as frequencies and percentages for categorical variables and as mean (±) standard deviation or median with interquartile range (IQR) for continuous variables. Nominal variables were analyzed using the chi-squared test or Fisher’s exact test, as appropriate, while continuous variables were analyzed using the Student’s *t*-test or Mann-Whitney U test. Statistical analyses were performed using SPSS version 29.0 (Armonk, NY: IBM Corp.).

## RESULTS

**Fig 1:**
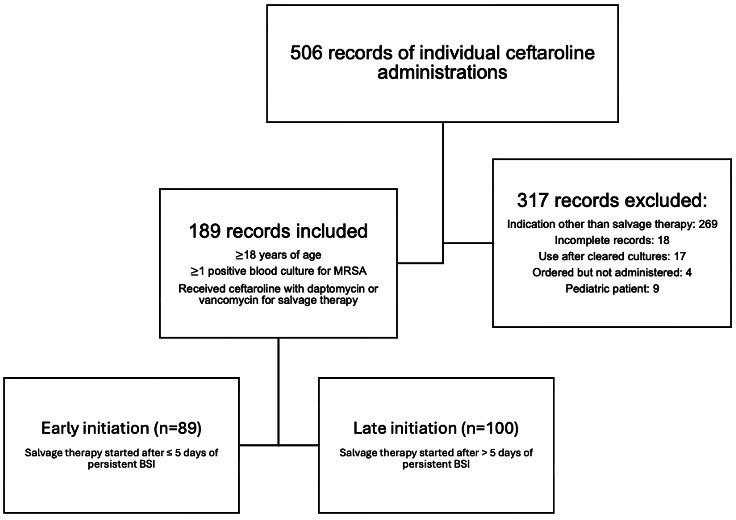
Overview of clinical inclusion and exclusion criteria for patients initially selected for the study due to ceftaroline use. Included patients were subsequently stratified into early (≤5 days) versus late (>5 days) initiation groups relative to persistent BSI.

Of the 506 patients identified via SlicerDicer who received ceftaroline within the study duration, 189 patients met the inclusion criteria (early initiation: 89 patients versus late initiation: 100 patients). Most excluded patients received ceftaroline for an indication other than salvage therapy (Fig. 1).

Overall, the patient characteristics were similar between the early and late initiation groups (Table 1). There was a higher median weight (71.0 kg versus 80.3 kg, *P* = 0.008) and percentage of patients with type II diabetes (25.8% versus 41.0%, *P* = 0.028) in the late initiation group. Conversely, there was a higher percentage of persons who inject drugs (PWID) in the early initiation group (36.0% versus 18.0%, *P* = 0.005). Renal function, as measured by median creatinine clearance (CrCl), was equivalent between groups (52.0 mg/dL vs 49.8 mg/dL, *P* = 0.582). Patient acuity, represented by the frequency of patients with a Pitt bacteremia score >4 and the percentage requiring ICU admission, was also comparable. There were no significant differences in representation by infection source, although the small cohort sizes limited our ability to draw conclusions. Notably, there were also comparable rates of intervention to pursue source control in both the early and late initiation populations (55.1% versus 58.0%, *P* = 0.684).

**TABLE 1:** Patient and bacteremia characteristics^*a*^^,*b*^

		Time to initiation of salvage therapy		
		≤5 days (*n* = 89)	>5 days (*n* = 100)	*P* value
Sex	Male	47 (52.8)	61 (61.0)	0.256
	Female	42 (47.2)	39 (39.0)	0.256
Age, years, median (IQR)		52.0 (32.0–64.0)	55.5 (43.0–67.8)	0.161
Weight, kg, median (IQR)		71.0 (62.1–88.7)	80.3 (68.0–99.6)	0.008
Race	Caucasian	58 (65.2)	57 (57.0)	0.251
	African American	28 (31.5)	39 (39.0)	0.279
Insured		72 (80.9)	86 (86.0)	0.344
Co-morbidities	Hypertension	42 (47.2)	55 (55.0)	0.284
	Hyperlipidemia	16 (18.0)	26 (26.0)	0.185
	Chronic kidney disease	26 (29.2)	35 (35.0)	0.396
	Type 2 diabetes	23 (25.8)	41 (41.0)	0.028
	Cirrhosis	4 (4.5)	4 (4.0)	>0.999
	Congestive heart failure	22 (24.7)	26 (26.0)	0.840
	Obesity	7 (7.9)	15 (15.0)	0.127
	HIV	3 (3.4)	1 (1.0)	0.344
	SOT/BMT	5 (5.6)	4 (4.0)	0.737
	COPD	14 (15.7)	12 (12.0)	0.457
	Malignancy on chemotherapy or immunomodulator	4 (4.5)	5 (5.0)	>0.999
	Hepatitis C	18 (20.2)	13 (13.0)	0.181
	PWID	32 (36.0)	18 (18.0)	0.005
CrCl, mg/dL, median (IQR)		52.0 (24.7–95.3)	49.8 (23.3–94.5)	0.582
Required ICU level care		57 (64.0)	67 (67.0)	0.669
Pitt bacteremia score ≥4		28 (31.5)	30 (30.0)	0.828
Bacteremia source	Skin and soft tissue	25 (28.1)	26 (26.0)	0.747
	Line-related	6 (6.7)	13 (13.0)	0.153
	Pulmonary	10 (11.2)	11 (11.0)	0.959
	Osteoarticular	10 (11.2)	16 (16.0)	0.343
	Endovascular	27 (30.3)	23 (23.0)	0.254
	Other	11 (12.4)	11 (11.0)	0.771
Source control pursued		49 (55.1)	58 (58.0)	0.684

^*a*^ HIV, human immunodeficiency virus; SOT, solid organ transplant; BMT, bone marrow transplant; COPD, chronic obstructive pulmonary disease; ICU, intensive care unit.

^*b*^ All data listed as value (%) unless otherwise specified.

Table 2 displays treatment characteristics between the patient groups. Approximately two-thirds of patients in the study were started on a daptomycin-based salvage regimen, with a similar distribution after stratification (61.8% versus 65.0%, *P* = 0.648). The remaining one-third were initiated on vancomycin plus ceftaroline. Nearly one in four patients in both the early and late groups required transition to an alternative salvage regimen from their initial combination (23.6% versus 24.0%, *P* = 0.948), which was decided at the individual physician’s discretion based on patient response to treatment and any adverse effects of antibiotic therapy. There was a non-significant trend showing a higher percentage of patients in the early initiation group receiving daptomycin dosed at 8 mg/kg q24h (23.6% versus 14.0%, *P* = 0.090). When comparing within cohorts, the percentage of patients that received 8 mg/kg (23.6%) versus 10 mg/kg (25.8%) was similar in the early initiation group, with a higher percentage of patients in the late group receiving 10 mg/kg dosing (29.0%) than those who received 8 mg/kg dosing (14.0%). Ceftaroline dosing was comparable between groups. Similarly, there was no significant difference in minimum inhibitory concentrations (MIC) or in the proportion of patients within therapeutic range on vancomycin based on trough data or AUC/MIC calculations. Infectious Diseases was consulted on all patients included in the study per internal policy. Finally, there was no difference in frequency of adverse effects related to salvage therapy in either group, with most patients having no adverse reaction.

**TABLE 2:** Treatment characteristics^*a*^^,*c*^

		Time to initiation of salvage therapy		
		≤5 days (*n* = 89)	>5 days (*n* = 100)	*P* value
Number of days until switched to salvage regimen, median (IQR)		4.0 (3.0–5.0)	8.0 (7.0–10.0)	<0.001
Initial regimen selected	DAP + CPT	55 (61.8)	65 (65.0)	0.648
	VAN + CPT	34 (38.2)	35 (35.0)	0.648
Need for second salvage regimen		21 (23.6)	24 (24.0)	0.948
Common dosage regimens	CPT 600 mg q8h	35 (39.3)	39 (39.0)	0.963
	CPT 600 mg q12h	16 (18.0)	21 (21.0)	0.601
	DAP 8 mg/kg q24h	21 (23.6)	14 (14.0)	0.090
	DAP 10 mg/kg q24h	23 (25.8)	29 (29.0)	0.628
MIC data	VAN MIC < 2 mg/L	89 (100.0)	100 (100.0)	>0.999
	VAN MIC ≤ 1 mg/L	63 (70.8)	71 (71.0)	0.974
	DAP MIC ≤ 1 mg/L (*n* = 49)	23 (100.0)	24 (92.3)	0.491
	CPT MIC ≤ 1 mg/L (*n* = 32)	15 (100.0)	17 (100.0)	>0.999
VAN monitoring	VAN AUC/MIC 400–600 (*n* = 27)^*b*^	11 (78.6)	11 (84.6)	>0.999
	VAN trough 10–20 mg/L (*n* = 114)	27 (47.4)	28 (49.1)	0.851
Infectious diseases consulted		89 (100.0)	100 (100.0)	>0.999
Adverse reactions to therapy	AKI	10 (11.2)	18 (18.0)	0.191
	Elevated CPK	2 (2.2)	6 (6.0)	0.285
	Neutropenia	3 (3.4)	2 (2.0)	0.668
	Hypersensitivity reaction	3 (3.4)	1 (1.0)	0.344
	Vancomycin infusion syndrome	1 (1.1)	1 (1.0)	>0.999
	Eosinophilic pneumonia	3 (3.4)	1 (1.0)	0.344
	*Clostridioides difficile* infection	2 (2.2)	2 (2.0)	>0.999
	None	70 (78.7)	71 (71.0)	0.228

^*a*^ DAP, daptomycin; CPT, ceftaroline; VAN, vancomycin; MIC, minimum inhibitory concentration; AUC, area under the curve; AKI, acute kidney injury; CPK, creatinine phosphokinase. All data listed as value (%) unless otherwise specified.

^*b*^ MICs were not available for all patients, vancomycin therapeutic drug monitoring (TDM*)* practices differed across facilities, and TDM information may have been incomplete for patients transferred from external institutions.

^*c*^ All data listed as value (%) unless otherwise specified.

Among patients who received dual therapy with ceftaroline, the timing of salvage therapy initiation did not affect 90-day all-cause mortality or hospital readmission rates, as shown in Table 3. Notably, early initiation (≤5 days) was associated with a significantly lower incidence of microbiologic recurrence than later initiation. Additional exploratory analyses of patients with infective endocarditis/cardiac abscess as their source of infection also demonstrated no significant difference in 90-day mortality rates between early versus late initiation cohorts, although there was a numerical trend in favor of initiation on or before day five (37.8% versus 52.2% respectively, *P* = 0.193).

**TABLE 3:** Comparison of primary endpoints^*a*^

	Time to initiation of salvage therapy		
	≤5 days (*n* = 89)	>5 days (*n* = 100)	*P* value
90-day all-cause mortality rate	33 (37.1)	43 (43.0)	0.407
90-day bacteremia recurrence rate	3 (3.4)	11 (11.0)	0.046
90-day hospital readmission rate	25 (28.1)	31 (31.0)	0.662

^*a*^ All data listed as value (%).

Early initiation of salvage therapy was not associated with a shorter interval from dual therapy onset to bacteremia clearance (Table 4). The median time to clearance after salvage therapy was 3.0 days in the early initiation group compared with 1.0 days in the late initiation group. In contrast, early initiation was associated with a shorter overall duration of bacteremia, a lower incidence of metastatic infection, and a reduced total duration of MRSA-directed therapy, while the duration of dual therapy was similar between the groups.

**TABLE 4:** Comparison of secondary endpoints^*a*^

	Time to initiation of salvage therapy		
	≤5 days (*n* = 89)	>5 days (*n* = 100)	*P* value
Days from combination therapy initiation to clearance (*n* = 175)	3.0 (1.0–5.0)	1.0 (0.0–5.0)	0.048
Total days bacteremic	7.0 (4.0–9.0)	10.5 (8.0–14.0)	<0.001
Evidence of metastatic disease, value (%)	55 (61.8)	82 (82.0)	0.002
Total treatment duration, days	46.0 (19.0–52.0)	50.0 (24.5–57.0)	0.013
Total days on second agent	11.0 (5.0–16.5)	12.0 (6.0–20.5)	0.442

^*a*^ All data listed as median (IQR) unless otherwise specified.

**Fig 2:**
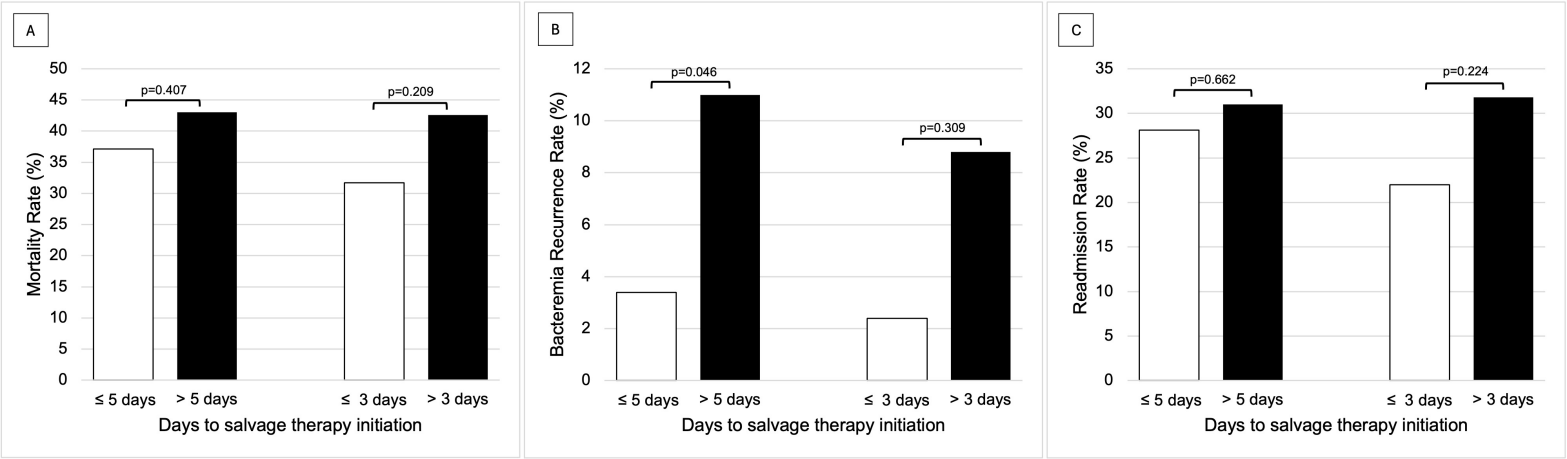
Comparison of primary endpoints, including 90-day (**A**) all-cause mortality rates, (**B**) bacteremia recurrence rates, and (**C**) all-cause hospital readmission rates, when salvage therapy was initiated at either ≤5 days or ≤3 days versus their later counterparts.

While the primary focus of this study was to assess outcomes in patients started on dual therapy on or before day 5 of MRSA BSI, an additional exploratory analysis assessing the primary outcomes for salvage therapy at ≤3 days or >3 days is shown in Fig. 2. For those who were transitioned to salvage therapy at ≤3 days of bacteremia, there was no statistically significant difference in 90-day mortality rates, although there appeared to be a larger difference than was seen with stratification at 5 days. There were also non-significant numerical trends toward reduced incidence of microbiologic recurrence and hospital readmission rates.

## DISCUSSION

There remains significant uncertainty surrounding the management of salvage therapy in persistent MRSA bacteremia. Ceftaroline has been proposed as a favorable agent for use in dual MRSA salvage therapy; however, the existing literature on ceftaroline has primarily focused on its comparative effectiveness versus standard monotherapy (13–15, 23). With the growing interest in its role as a salvage agent, there is a critical need for evidence defining the optimal timing of initiation when ceftaroline is employed for this use. Within our retrospective, multi-center hospital network, early initiation of ceftaroline-based combination therapy was associated with a modest trend toward reduced mortality and a statistically significant reduction in bacteremia recurrence compared to later initiation, although 90-day all-cause mortality rates and hospital readmission rates did not differ significantly. These findings suggest a potential benefit with earlier initiation than the current guideline suggestion of 7 days (8).

On evaluation of our population’s bacteremia characteristics, the early initiation group exhibited a longer median time to culture clearance after ceftaroline addition (3.0 days versus 1.0 day), likely due to confounding from the inclusion of patients transferred for procedural management and source control. However, total bacteremia duration was shorter with earlier salvage therapy (7.0 days versus 10.5 days), consistent with prior research demonstrating reduced duration when started on daptomycin plus ceftaroline within 72 h of bacteremia onset (14). The shorter bacteremia duration in the early initiation group was reflected by lower rates of metastatic disease (61.8% versus 82.0%) and a shorter total antibiotic course (46 days versus 50 days), which may be reflective of the shorter duration of bacteremia and lower burden of metastatic infection. These findings suggest that earlier initiation of combination salvage therapy may shorten the duration of MRSA bacteremia and reduce downstream complications, including metastatic infections, prolonged hospital stay, and increased mortality associated with persistent *Staphylococcus aureus* bacteremia (SAB) beyond 72 h (3).

Another area of uncertainty in the literature is the optimal duration of salvage therapy following initiation, as there is limited guidance regarding the appropriate duration of the secondary agent (24). Prior data indicate no additional benefit from extending combination therapy beyond 7 days after culture clearance (25). Other investigators have advocated an approach involving early intensive salvage therapy with subsequent de-escalation to monotherapy after stabilization and culture clearance, thereby minimizing overall antibiotic exposure (2). We found no significant difference in the total duration of dual therapy between cohorts in our study (11.0 versus 12.0 days), suggesting that the timing of salvage initiation did not influence physicians’ decisions regarding the overall duration of dual therapy. Future research should aim to investigate the appropriate duration of salvage therapy that balances therapeutic efficacy with the risk of adverse effects, such as hematologic abnormalities, rash, and renal dysfunction (26, 27). We observed a similar incidence of these adverse effects in both the early and late initiation groups, likely reflecting the similar median duration of combination therapy observed in both cohorts. The shorter total duration of antibiotic therapy observed in the early initiation group may hypothetically reduce the risk of adverse effects associated with cumulative exposure, although such preventative outcomes are inherently unquantifiable (28).

Due to prior evidence of mortality benefit and faster clearance with switching to dual therapy within 72 h of bacteremia onset, we conducted a subgroup analysis stratifying patients by the timing of ceftaroline addition (≤3 days versus >3 days of persistent BSI) (13, 14). Although not statistically significant, the numeric trends toward reduced mortality, recurrence, and readmission rates align with prior data demonstrating increased mortality risk after 3 days of persistent SAB (3). The lower mortality trend observed with more aggressive escalation to salvage therapy suggests potential benefits as early as day 3 of persistent BSI. However, additional studies with a more robust sample size of patients receiving dual therapy within ≤3 days are needed to validate these findings.

Prior studies have largely evaluated ceftaroline in combination with either vancomycin or daptomycin, although there has been some limited research selecting for both dual therapy strategies (13–15, 17, 23). A strength of our study is that it represents a mixed population, with approximately two-thirds of the patients receiving a daptomycin-based salvage regimen, while the remaining one-third received vancomycin plus ceftaroline. This may be explained by the institutional preference to start on vancomycin and change to daptomycin while adding ceftaroline if persistently bacteremic. In practice, dual therapy implementation requires an individualized approach, resulting in the use of both daptomycin- and vancomycin-based regimens, thus making the results of our study more generalizable. This is evidenced by nearly one in four patients in the study requiring an alternative salvage regimen after their initial dual therapy selection. Additional research with a more direct comparison of the degree of impact of early initiation in daptomycin versus vancomycin use with ceftaroline for salvage therapy would also be of value.

This retrospective cohort study had several important limitations. To start, it is difficult to ascertain why certain patients in the study were chosen to receive earlier combination therapy and how this may have influenced the data given the retrospective nature of the study. The decision was made at the discretion of the individual physician and may have been influenced by non-objectifiable reasons. Variability in vancomycin monitoring practices, including AUC/MIC- and trough-based approaches, must also be acknowledged and likely stems from the lack of implementation among satellite institutions.

Baseline demographics and comorbidities remained largely the same across both cohorts, with significant differences observed only in body weight, type II diabetes, and the proportion of PWID. Among these variables, earlier initiation of salvage therapy in PWID may reflect implicit provider-level bias. However, as the flagship institution is a tertiary center that accepts transfers, this observation may also be confounded by the transfer of patients with persistent bacteremia from outside facilities. Alternatively, perhaps concern for an increased risk of endocarditis in PWID and the associated worse clinical outcomes may have resulted in more aggressive antibiotic therapy up front (29). Despite this, the proportion of patients with a Pitt bacteremia score ≥4 and those requiring ICU admission were equivalent between the groups, suggesting a similar acuity of illness between the two patient populations.

Additional constraints associated with a retrospective study design must be acknowledged. Data collection was limited within the confines of prior documentation or record availability. This was most noticeable in transfers from outside facilities, occasionally resulting in patient exclusion due to inability to confirm treatment details. Furthermore, the lack of randomization results in biases associated with observational studies. Although study cohorts were largely comparable across anticipated confounding variables, unmeasured variables may still have influenced outcomes. Furthermore, data collection was constrained to variables documented in the electronic medical record and defined *a priori* in the standardized abstraction tool; as such, potential clinically relevant variables may not have been captured.

Inability or decision to not pursue source control raises another limitation, as source control remains a pillar of management for persistent MRSA bacteremia (3, 8). The timing of when this was pursued could have had an unknown influence on outcomes. However, the rates of source control intervention were similar between both groups, with approximately half of the patients in both study populations not undergoing source control. Even with many patients not receiving source control, there remains evidence of benefit from early initiation of salvage therapy.

### Conclusion

This study represents one of the largest cohorts to date examining patients receiving dual salvage therapy for MRSA bacteremia with ceftaroline-based regimens (13–15, 17, 23). Although earlier initiation of salvage therapy (≤5 or ≤3 days) was not associated with a statistically significant reduction in mortality, there was a significant reduction in bacteremia recurrence if salvage therapy was started within 5 days. There was also a non-significant trend observed toward lower mortality and readmission rates with early intervention. Furthermore, earlier initiation was associated with fewer total days of bacteremia, shorter overall duration of antibiotic therapy, and lower incidence of metastatic disease, suggesting the potential for clinical benefit with more aggressive initiation of salvage therapy. Moving forward, larger prospective randomized studies would be advantageous to further evaluate duration of combination therapy and whether the benefits seen with early escalation to dual therapy allow for shorter treatment duration or for a safe transition to oral antibiotics to complete therapy.

### Summary

There were no differences in mortality or readmission rates with earlier initiation of ceftaroline-based dual therapy for MRSA bacteremia; however, earlier initiation was associated with a shorter duration of bacteremia, lower recurrence, less metastatic disease, and a shorter duration of therapy with earlier initiation.

## Data Availability

The data that support the findings of this study are available upon reasonable request from the corresponding author.
